# Signaling mediated by the NF-κB sub-units NF-κB1, NF-κB2 and c-Rel differentially regulate *Helicobacter felis*-induced gastric carcinogenesis in C57BL/6 mice

**DOI:** 10.1038/onc.2013.334

**Published:** 2013-08-26

**Authors:** M D Burkitt, J M Williams, C A Duckworth, A O'Hara, A Hanedi, A Varro, J H Caamaño, D M Pritchard

**Affiliations:** 1 Department of Gastroenterology, The Henry Wellcome Laboratories, Institute of Translational Medicine, University of Liverpool, Liverpool, UK; 2 Department of Cellular and Molecular Physiology, Institute of Translational Medicine, University of Liverpool, Liverpool, UK; 3 IBR-School of Immunity and Infection, College of Medical and Dental Sciences, University of Birmingham, Birmingham, UK

**Keywords:** *Helicobacter felis* infection, NF-κB, MALT lymphoma, gastric cancer

## Abstract

The classical nuclear factor-kappaB (NF-κB) signaling pathway has been shown to be important in a number of models of inflammation-associated cancer. In a mouse model of *Helicobacter*-induced gastric cancer, impairment of classical NF-κB signaling in the gastric epithelium led to the development of increased preneoplastic pathology, however the role of specific NF-κB proteins in *Helicobacter-*associated gastric cancer development remains poorly understood. To investigate this C57BL/6, *Nfkb1*^*−/−*^*, Nfkb2*^*−/−*^ and *c-Rel*^*−/−*^ mice were infected with *Helicobacter felis* for 6 weeks or 12 months. Bacterial colonization, gastric atrophy and preneoplastic changes were assessed histologically and cytokine expression was assessed by qPCR. *Nfkb1*^*−/−*^ mice developed spontaneous gastric atrophy when maintained for 12 months in conventional animal house conditions. They also developed more pronounced gastric atrophy after short-term *H. felis* colonization with a similar extent of preneoplasia to wild-type (WT) mice after 12 months. *c-Rel*^*−/−*^ mice developed a similar degree of gastric atrophy to WT mice; 3 of 6 of these animals also developed lymphoproliferative lesions after 12 months of infection. *Nfkb2*^*−/−*^ mice developed minimal gastric epithelial pathology even 12 months after *H. felis* infection. These findings demonstrate that NF-κB1- and NF-κB2-mediated signaling pathways differentially regulate the epithelial consequences of *H. felis* infection in the stomach, while c-Rel-mediated signaling also appears to modulate the risk of lymphomagenesis in gastric mucosa-associated lymphoid tissue.

## Introduction

In many malignancies, changes in the tissue microenvironment, influenced by immune responses, create a niche that promotes selection of malignant clones.^1^ *Helicobacter pylori-*associated gastric cancer is an example of an inflammation-associated cancer in which both the immunological trigger and the pathological sequence leading to cancer development have been defined. Gastric colonization with *Helicobacter* species leads to a stereotypical pathological sequence in which superficial gastritis progresses to atrophic gastritis, metaplasia, dysplasia and eventually cancer over a prolonged period in both humans^2^ and rodent models.^3^

Despite a worldwide prevalence of *H. pylori* infection of at least 50%,^4^ gastric cancer remains relatively rare (peak incidence, in men from Eastern Asia, 42.4 per 100 000^5^). This demonstrates that factors other than *H. pylori* infection contribute to the development of gastric cancer, and several lines of evidence have suggested that immune responses are important regulators of *Helicobacter-*induced disease progression.

A meta-analysis of 71 case–control studies has revealed complex relationships between polymorphisms in inflammatory cytokines and their receptors and gastric cancer risk. Overall, however, a pro-inflammatory genotype appears to enhance an individual's risk of developing gastric cancer when infected with *H. pylori*.^6^ Transgenic animal studies have also revealed complex associations between Th1 cytokines and gastric carcinogenesis. Overexpression of interleukin-1β (IL-1β) was sufficient to promote murine carcinogenesis in the absence of *Helicobacter* infection,^7^ while overexpression of interferon-γ (IFN-γ) in parietal cells inhibited progression of *Helicobacter felis-*induced gastric carcinogenesis.^8^

These studies emphasize the importance of the host inflammatory response in gastric carcinogenesis, but also raise questions regarding the regulatory mechanisms through which inflammatory cytokines may generate a pro-carcinogenic environment. The nuclear factor-kappaB (NF-κB) signaling pathways orchestrate inflammatory responses. Signaling through these pathways is commonly separated into classical and alternative arms, with distinct triggers and transcriptional targets. The classical NF-κB activation pathway is typified by signaling via dimers of RelA and NF-κB1, but can also encompass hetero-dimers of these proteins with c-Rel. The activation of this pathway is triggered by diverse stimuli including the binding of cytokines such as tumor necrosis factor α (TNFα) and IL-1β to their receptors. This leads to the proteasomal degradation of IκBα under the influence of inhibitor of κB kinase-β. Degradation of IκBα allows the nuclear translocation of the NF-κB protein dimer, resulting in DNA binding and subsequent transcriptional activity.^9^

The alternative pathway of NF-κB signaling involves RelB-NF-κB2 dimers. NF-κB2 is synthesized as a 100-kDa precursor protein (NF-κB2 p100) with a C-terminal ankyrin repeat domain that inhibits nuclear localization. Binding of BAFF, CD40L or lymphotoxin αβ to their receptors result in phosphorylation of NF-κB2 under the control of IKK-α and NIK, leading to partial degradation of NF-κB2. This exposes the nuclear translocation domain of NF-κB2 and the Relb-NF-κB2 dimer translocates to the nucleus where it regulates transcription.^9^ In addition to these established pathways of NF-κB activation, there is also increasing interest in both hybrid NF-κB pathways, for instance, recent data have demonstrated a novel mechanism for NF-κB P100 activation of classical pathway dimers and the potential for cross talk both between the NF-κB pathways and other signaling pathways.^10^

Transgenic mouse studies investigating the effects of *Ikkb* deletion have established a role for classical NF-κB signaling in inflammation-associated carcinogenesis in several parts of the GI tract.^11, 12^ In the stomach, *Ikkb* has been targeted for deletion under the control of the FoxA3 promoter; following *H. felis* infection, these mice developed more severe gastric atrophy, and progressed more rapidly to gastric preneoplasia than control animals.^12^ These results demonstrate that signaling via the classical NF-κB-activation pathway can modulate risk of inflammation-associated carcinogenesis, but neither the effects of the alternative NF-κB-signaling pathway nor the roles of specific NF-κB proteins in gastric carcinogenesis have been defined.

Other transgenic mouse studies indicate that NF-κB2-mediated signaling might have a role in gastric epithelial homeostasis and structure. Mice with a C-terminal truncation of *Nfkb2* (leading to enhanced DNA binding of RelB/NF-κB2 P52 dimers) develop massive gastric hyperplasia and gastric outlet obstruction.^13^ There is also *in-vitro* evidence that *H. pylori* can enhance NF-κB2 DNA binding in B lymphocytes.^14^ Hence, a *Helicobacter-*induced model of gastric carcinogenesis appears to be an ideal model to investigate whether the NF-κB2-mediated signaling influences inflammation-associated carcinogenesis.

To investigate the role of specific classical and alternative pathway members in gastric carcinogenesis, we have used transgenic mice with germline deletions of *Nfkb1*, *Nfkb2* or *c-Rel* and the C57BL/6 wild-type (WT) mouse/*H. felis* infection model. In this model, the gastric antrum is reliably colonized with *H. felis*, and pathology develops in the gastric corpus 6 weeks after infection^15^ when WT mice begin to develop parietal cell loss. More advanced premalignant lesions develop in the gastric corpus over ∼12–15 months, but advanced cancers are rare.^16^ In this model an increased severity of gastric pathology in females has been reported,^17^ hence our experiments have been performed exclusively in female mice.

## Results

### NF-κB1 deletion leads to spontaneous gastric inflammation and increased cellular turnover

**Figure 1:**
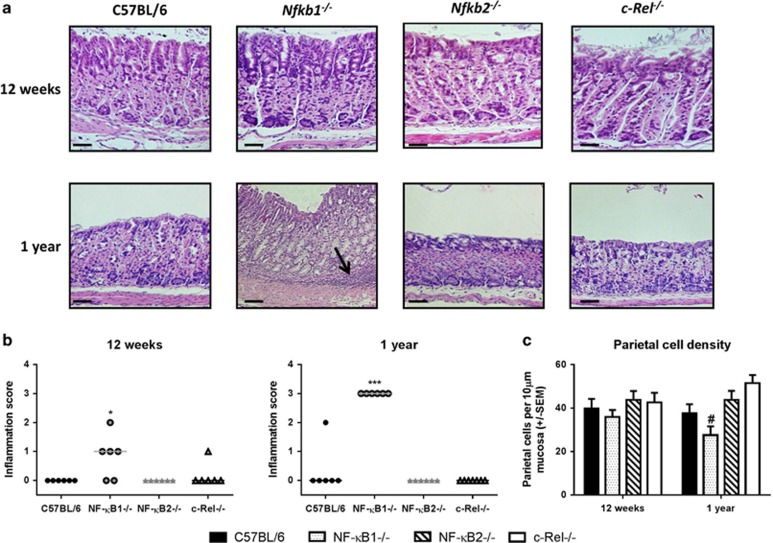
(**a**) Representative photomicrographs: gastric corpus from C57BL/6, *Nfkb1*^*−/−*^, *Nfkb2*^*−/−*^ and *c-Rel*^*−/−*^ mice aged 12 weeks or 1 year. Scale bar 50 μm. (**b**) Visual analog scoring of inflammatory cell infiltration into gastric corpus, 12-week- and 1-year-old mice (individual scores and median score plotted). (**c**) Parietal cell density, gastric corpus of 12-week-old and 1-year-old mice. (**d**) Relative mRNA abundance of TNF-α, IL-1β and IFN-γ, gastric corpus of 12-week-old mice. *P<0.05, ***P<0.001 by tests stated in text, ^#^significant difference compared with (*cf*) WT mice at the same time-point, *P*<0.05.

As spontaneous autoimmune gastric corpus atrophy is an independent risk factor for human gastric carcinogenesis,^18^ we examined the gastric corpus of WT, *Nfkb1*^*−/−*^*, Nfkb2*^*−/−*^ and *c-Rel*^*−/−*^ mice aged 12 weeks and 1 year to establish whether there was any evidence of spontaneous gastric pathology. Tissues were taken for histology from groups of at least 6 female mice at both time-points and for protein and nucleic acids at 12 weeks. Figure 1a shows representative photomicrographs of the gastric corpus of animals of each genotype at these time-points. At 12 weeks, minimal inflammation was present in all groups and quantitative scoring showed no significant differences between genotypes (Figure 1b), but did suggest a trend towards increased inflammation in *Nfkb1*^*−/−*^ mice (median score of 1 *cf* 0 in all other groups). By 12 months, *Nfkb1*^*−/−*^ mice had significantly higher inflammatory cell infiltrate scores than other groups with coalescing inflammatory cell infiltrates extending into the submucosa (arrowed in Figure 1a).

To determine whether *Nfkb1*^*−/−*^ mice also developed spontaneous gastric atrophy, we quantified mean mucosal thickness and parietal cell number. When parietal cell density was plotted (number of parietal cells per 10 μm of mucosal thickness), 12-month-old *Nfkb1*^*−/−*^ mice showed significantly lower scores than similarly aged WT mice, suggesting the presence of some atrophic gastritis (Figure 1c).

**Figure 2:**
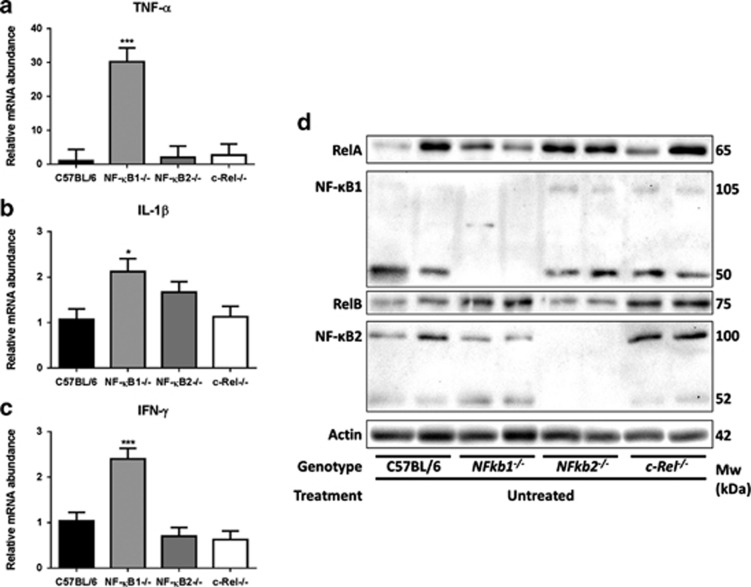
(**a**–**c**) Relative mRNA abundance of Th1 cytokines ((**a**) TNF-α (**b**) IL-1β, (**c**) IFN-γ): gastric corpus, 12-week-old mice infected with *H. felis* *P<0.05, ***P<0.005. (**d**) Western blots demonstrating expression of RelA, NF-κB1, RelB and NF-κB2 in lysates derived from gastric corpus lysates of 12-week-old WT, *Nfkb1*^*−/−*^, *Nfkb2*^*−/−*^ and *c-Rel*^*−/−*^ mice.

To assess whether the observed spontaneous inflammatory response was associated with any change in cytokine profile, we assayed Th1 cytokines in the gastric corpus of 12-week-old mice. *Nfkb1*^*−/−*^ mice showed a 30-fold increase in mRNA levels of TNF-α as well as small increases in transcript abundance of IL-1β and IFN-γ compared with their WT counterparts (Figure 2a and c).

To determine whether this spontaneous inflammatory phenotype correlated with altered baseline NF-κB signaling we determined the abundance of NF-κB sub-units by western blotting of protein lysates from the gastric corpus of untreated mice. Unfortunately, owing to limitations of antibody specificity, we were unable to quantify the abundance of c-Rel in these lysates. In keeping with previous studies using mice of these genotypes,^19, 20^ no major consistent differences in expression of other NF-κB proteins were demonstrated in the gastric corpus mucosa of unstimulated animals with transgenic deletion of one NF-κB sub-unit (Figure 2d).

### NF-κB1- and NF-κB2-mediated signaling pathways differentially influence microbial colonization and inflammatory responses to short-term *H. felis* infection

**Figure 3:**
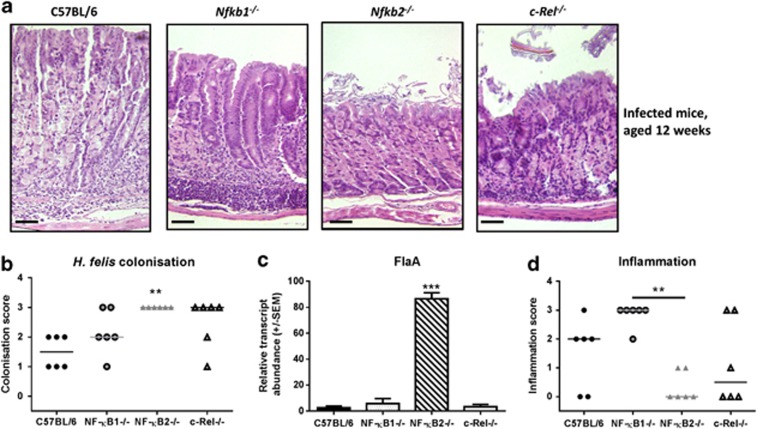
(**a**) Representative photomicrographs: gastric corpus of mice infected with *H. felis* for 6 weeks. Scale bar 50 μm. (**b**) Visual analog scoring of *H. felis* colonization: gastric antrum of mice infected with *H. felis* for 6 weeks, individual scores and median plotted, **P<0.01. (**c**) Relative *H. felis* FlaA DNA abundance in gastric antrum of mice infected with *H. felis* for 6 weeks, ***P<0.001. (**d)** Visual analog scoring of inflammatory cells infiltrating gastric corpus: mice infected with *H. felis* for 6 weeks, individual scores and median score plotted, **P<0.01.

Having demonstrated that *Nfkb1*^*−/−*^ mice developed spontaneous gastritis with mucosal atrophy, we investigated the effects of short-term *H. felis* colonization on the gastric mucosa of mice with specific NF-κB sub-unit deletions. Groups of 6-week-old WT, *Nfkb1*^*−/−*^, *Nfkb2*^*−/−*^ and *c-Rel*^*−/−*^ mice were infected with *H. felis* for 6 weeks. Colonization was confirmed by microscopy with severity scored by visual analog scale. Analysis of the different mutant strains showed that *Nfkb2*^*−/−*^ mice had higher colonization scores than WT mice (*P*<0.05, Kruskal–Wallis test, Figure 3b). This was confirmed by quantitative PCR for *H. felis-*specific FlaA, which was 30-fold more abundant in the gastric antrum of *H. felis-*infected *Nfkb2*^*−/−*^ mice than WT mice (one-way analysis of variance (ANOVA), *P*<0.001; Figure 3c).

**Figure 4:**
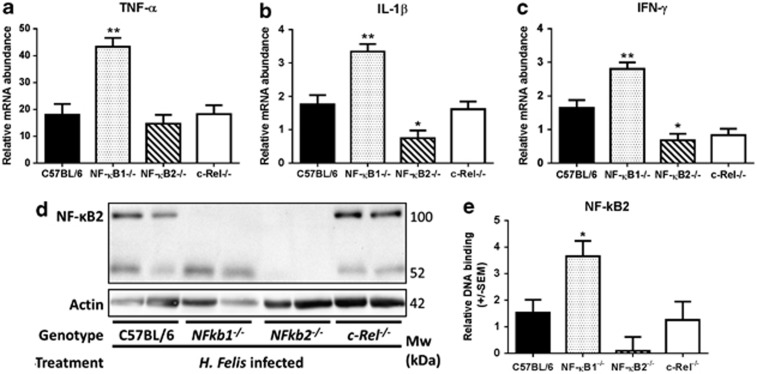
(**a**–**c**) Relative mRNA abundance of Th1 cytokines ((**a**) TNF-α (**b**) IL-1β (**c**) IFN-γ): gastric corpus, 12-week-old mice infected with *H. felis* *P<0.05, **P<0.01. (**d**) Western blot demonstrating expression of NF-κB2 in gastric corpus of WT, *Nfkb1*^*−/−*^, *Nfkb2*^*−/−*^ and *c-Rel*^*−/−*^ mice infected with *H. felis* for 6 weeks. (**e**) DNA-binding enzyme-linked immunosorbent assay (ELISA) demonstrating relative DNA binding of NF-κB2 p52 in gastric mucosa of WT, *Nfkb1*^*−/−*^, *Nfkb2*^*−/−*^ and *c-Rel*^*−/−*^ mice infected with *H. felis* for 6 weeks, *P<0.05.

In the corpus of *Helicobacter* colonized mice (Figure 3d), there was a trend towards higher inflammation scores than WT mice in *Nfkb1*^*−/−*^ mice and a trend towards lower scores than WT in *Nfkb2*^*−/−*^ animals. These differences did not reach statistical significance; however, *Nfkb1*^*−/−*^ mice did show significantly more severe inflammatory cell infiltrates than *Nfkb2*^*−/−*^ mice (Figure 3a). These differences in inflammatory burden in *Nfkb1*^*−/−*^ and *Nfkb2*^*−/−*^ mice were also reflected in the abundance of Th1 cytokine transcripts (Figures 4a and c). In *Nfkb1*^*−/−*^ mice, TNF-α transcripts were upregulated 2.4-fold compared with infected WT mice, while IL-1β and IFN-γ were upregulated by factors of 1.9 and 1.7, respectively. In contrast, *H. felis-*infected *Nfkb2*^*−/−*^ mice had an attenuated Th1 cytokine response with significantly lower IL-1β (2.3-fold) and IFN-γ (2.4-fold) transcript abundance compared with infected WT mice.

As deletion of NF-κB2 attenuated the phenotype associated with acute *H. felis* infection the function of this sub-unit was investigated in more detail. Upstream regulation of NF-κB2 signaling by NIK was investigated by western blotting, but abundance of NIK was low, and no significant differences were observed in WT mice following *H. felis* infection (Supplementary Figure 1). In contrast NF-κB2 p100 was fully processed to p52 in the gastric corpus of *Nfkb1*^*−/−*^ mice following infection (Figure 4d), this was also associated with a significant increase in DNA binding of this sub-unit in infected *Nfkb1*^*−/−*^ mice compared with infected WT mice (Figure 4e).

### NF-κB1- and NF-κB2-mediated signaling pathways differentially regulate the development of acute gastric atrophy following 6 weeks *H. felis* infection

**Figure 5:**
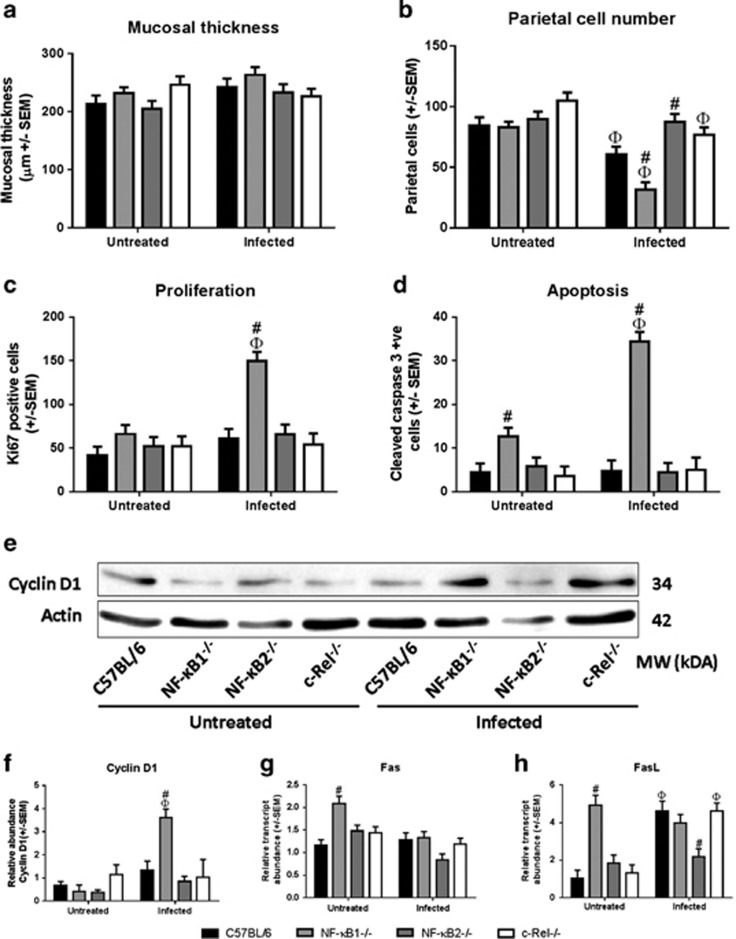
(**a**) Gastric corpus mucosal thickness: mice untreated and infected with *H. felis*. (**b)** Number of parietal cells scored in gastric corpus mucosa: mice untreated or infected with *H. felis*. ^Φ^Significant difference *cf* untreated mice of same genotype, *P*<0.05. ^#^Significant difference *cf* WT receiving same treatment, *P*<0.05. (**c**) Ki67-positive cells in gastric corpus: mice untreated and infected with *H. felis* for 6 weeks. (**d)** Cleaved caspase-3-positive cells in gastric corpus: mice untreated or infected with *H. felis* for 6 weeks. (**e**, **f)** Western blot and densitometry for cyclin D1 in untreated and infected mice aged 12 weeks. (**g**, **h**) relative mRNA abundance of Fas and FasL, respectively, in gastric corpus tissue of mice either untreated or infected with *H. felis* for 6 weeks. ^Φ^Significant difference *cf* untreated mice of same genotype, *P*<0.05. ^#^Significant difference *cf* WT receiving same treatment, *P*<0.05.

Following short-term *H. felis* infection, mucosal thickness was unaltered (Figure 5a), but differences in parietal cell number were observed (Figure 5b): 29% fewer parietal cells were detected in WT mice following infection (*P*<0.05, two-way ANOVA). *Nfkb1*^*−/−*^ mice showed enhanced gastric atrophy, with 62% parietal cell loss (*P*<0.05).

Despite high levels of *H. felis* colonization, *Nfkb2*^*−/−*^ mice appeared to be protected against the development of gastric mucosal pathology, exhibiting only 3% parietal cell loss at this time-point. These observations suggest that deletion of NF-κB1 or NF-κB2 have contrasting effects on gastric epithelial responses to *H. felis* infection.

To determine whether these changes in gastric morphology resulted from altered epithelial cell turnover, we quantified proliferating (Ki67) and apoptotic (cleaved caspase-3) cells by immunohistochemistry. No differences were observed in cell turnover between WT mice and either *c-Rel*^*−/−*^, or *Nfkb2*^*−/−*^ mice, but differences were observed between WT and *Nfkb1*^*−/−*^ mice. Untreated WT mice had a proliferation index of 41.2 cells per section, which did not change significantly after *H. felis* infection. Although baseline cell proliferation in *Nfkb1*^*−/−*^ mice was similar to WT mice, *H. felis* infection resulted in a 2.5-fold increase in the number of proliferating cells in this mutant strain (*P*<0.05, two-way ANOVA; Figure 5c). This difference was confirmed by western blotting which showed significantly increased cyclin D1 abundance in infected *Nfkb1*^*−/−*^ mice (*P*<0.05, two-way ANOVA and HS test; Figures 5e and f).

Differences were also observed in the number of apoptotic events observed in the gastric corpus of NF-κB1^*−/−*^ compared with WT mice. Untreated *Nfkb1*^*−/−*^ mice had an apoptotic index 2.8-fold higher than WT mice, which increased 2.7-fold further following *H. felis* infection (*P*<0.05, two-way ANOVA; Figure 5d). Differences in baseline apoptotic index may reflect differences in the abundance of mRNAs for the pro-apoptotic proteins Fas and FasL, which were upregulated 1.8- and 4.8-fold, respectively, in untreated *Nfkb1*^*−/−*^ compared with WT mice. However, after *H. felis* infection, the abundance of Fas and FasL transcripts was similar in both WT and *Nfkb1*^*−/−*^ animals (*P*<0.05, two-way ANOVA, Figures 5g and h), suggesting that an alternative mechanism may be responsible for the increase in apoptosis seen in *Nfkb1*^*−/−*^ mice after *H. felis* infection.

### Classical and alternative NF-κB signaling pathways differentially regulate the outcomes of chronic *H. felis* infection

To establish whether the differences in inflammation, cell turnover and atrophy observed in mice after short-term *H. felis* infection had long-term sequelae, we performed long-term *Helicobacter* infections. Groups of at least six female mice were infected with *H. felis* at 6 weeks and maintained for 1 year alongside uninfected controls. Quantitative histological assessment was performed for gastric antral colonization with *Helicobacter*, gastric corpus inflammation, mucosal thickness and parietal cell numbers as previously described and morphology was assessed by a board accredited veterinary pathologist (JW) with quantification of specific features. These findings are summarized in Supplementary Table 1.

During long-term *H. felis* infection, two animals died before 1 year. Both were *H. felis* colonized *Nfkb2*^*−/−*^ mice, one 6 months and one 8 months after *Helicobacter* administration. Necropsy demonstrated marked ascites, and histological examination showed renal lesions in both mice. *H. felis* colonization of the stomach was confirmed histologically, and the gastric corpus mucosa did not show significant pathological changes. They were excluded from further analyses due to their intercurrent renal disease and reduced duration of *H. felis* colonization.

**Figure 6:**
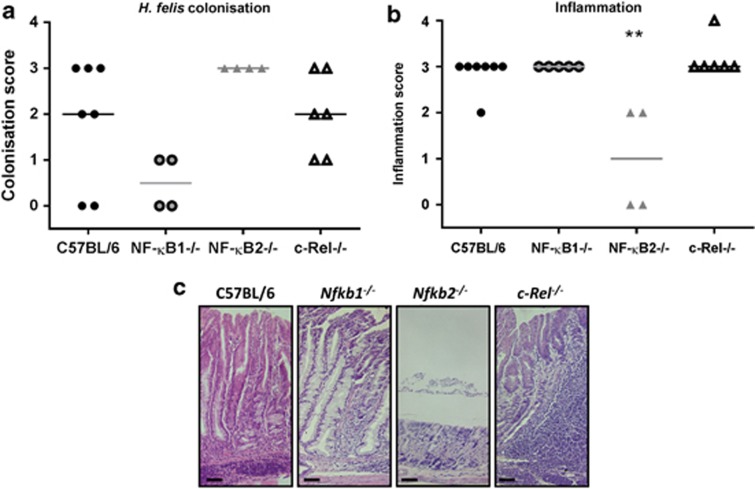
(**a**, **b**) Visual analog scoring of gastric mucosa from mice infected with *H. felis* for 1 year, individual scores and median plotted. (**a**) Antral *H. felis* colonization scores. (**b**) Corpus inflammatory cell infiltration scores. **P<0.05. (**c**) Representative photomicrographs: gastric corpus mucosa from C57BL/6, *Nfkb1*^*−/−*^, *Nfkb2*^*−/−*^ and *c-Rel*^*−/−*^ mice following *H. felis* infection for 1 year. Scale bar 50 μm.

After 1 year, no statistical differences were demonstrated in the *Helicobacter* colonization scores of mice of different genotypes (Figure 6a), however, two WT and two *Nfkb1*^*−/−*^ mice had no visible *Helicobacter* organisms in the gastric antrum section examined. After unblinding, the gastric corpus of these mice was assessed for *Helicobacter-*induced pathology. In each of the four mice without visible *H. felis* organisms, there was profound gastric corpus atrophy and more advanced preneoplastic lesions were seen, suggesting that the *Helicobacter* niche had been diminished or lost as a consequence of *H. felis-*induced gastric hypochlorhydria.

**Figure 7:**
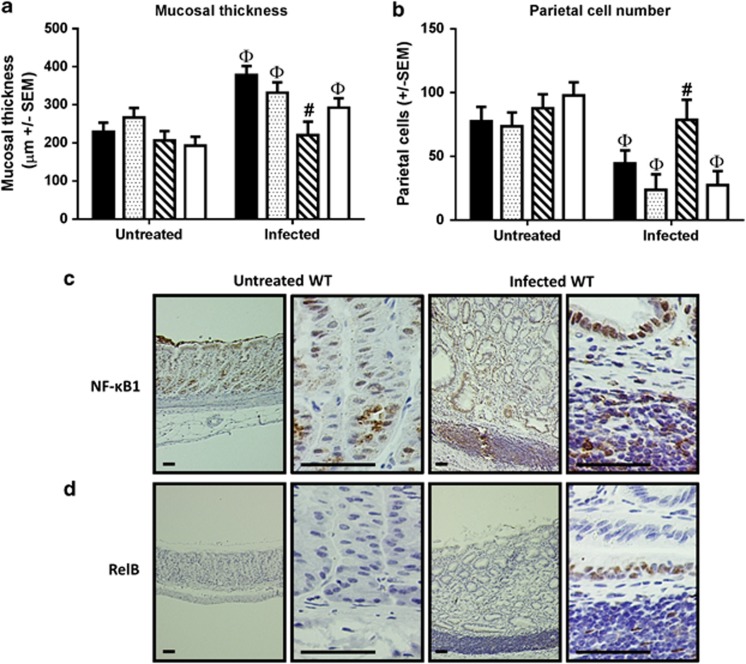
(**a**) Gastric corpus mucosal thickness: 1-year-old mice untreated or infected with *H. felis*. (**b**) Gastric corpus parietal cell numbers: 1-year-old mice untreated or infected with *H. felis*. ^Φ^significant difference *cf* untreated mice of same genotype, *P*<0.05. ^#^marks significant difference *cf* WT receiving same treatment, *P*<0.05. (**c**) Representative photomicrographs demonstrating NF-κB1 immunostaining in gastric corpus of 1-year-old untreated WT mouse (left two panels), and WT mouse infected with *H. felis* for 12 months (right panels). (**d**) Representative photomicrographs demonstrating RelB immunostaining in corpus of 1-year-old untreated WT mouse (left two panels), and WT mouse infected with *H. felis* for 12 months (right panels). Scale bar 50 μm.

Representative photomicrographs of the epithelial pathology induced by chronic *H. felis* infection in the gastric corpus are shown in Figure 6c. Inflammatory cell infiltrates were seen in mice of all genotypes after colonization with *H. felis* for 1 year; however, the scores in *Nfkb2*^*−/−*^ mice were significantly lower than those from all other groups (median score 1 *cf* 3 for all other groups) (*P*<0.01, Kruskal–Wallis test; Figure 6b). Pronounced gastric atrophy was observed in WT mice infected with *H. felis* for 1 year: gastric corpus mucosa was significantly thickened (1.65-fold) and 43% fewer parietal cells were present in *H. felis-*infected WT mice compared with age-matched uninfected animals (*P*<0.05, two-way ANOVA; Figures 7a and b). *Nfkb1*^*−/−*^ mice and *c-Rel*^*−/−*^ mice had equivalent degrees of mucosal atrophy to WT mice. In contrast, *Nfkb2*^*−/−*^ mice continued to have low gastric inflammation scores and did not develop significant gastric mucosal thickening or parietal cell loss.

To determine whether NF-κB sub-units were expressed differently in mice that had undergone long-term *H. felis* infection, immunohistochemical staining was performed. Due to antibody limitations only NF-κB1 and RelB could reliably be detected in formalin fixed, paraffin-embedded tissue samples. In 1-year-old WT mice nuclear localized NF-κB1 was detected in all visualized gastric glands, with stronger staining being observed in chief cells and mucous cells towards the luminal surface of the gastric gland. In mice that had been infected with *H. felis* for 12 months, nuclear localization of NF-κB1 was almost ubiquitous with similar intensity of staining being observed in all cell types throughout glands. In addition to the strong nuclear staining seen in epithelial tissues the inflammatory cell infiltrates of these animals also strongly expressed nuclear NF-κB1 (Figure 7c). In contrast, and confirming antibody specificity, this antibody did not immunostain the gastric mucosa of *Nfkb1*^*−/−*^ mice.

In 1-year-old untreated mice, very few RelB-positive cells were observed. Following *H. felis* infection for 1 year, however, RelB was expressed in all observed lymphoid follicles, with ∼50% of the cells in these follicles expressing the antigen. RelB was also observed in occasional epithelial glands, either as an isolated, morphologically metaplastic, gland expressing the antigen or in close association with a lymphoid follicle (Figure 7d).

**Figure 8:**
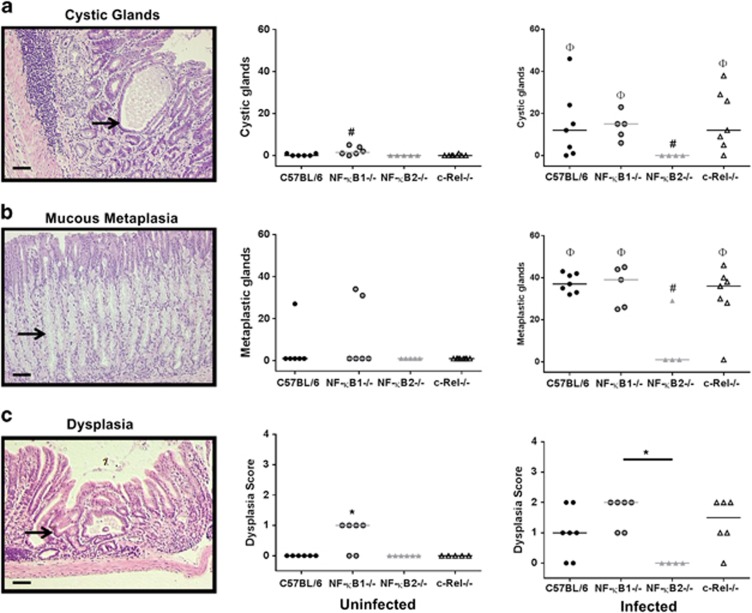
Photomicrographs and quantitative scoring of epithelial pathology in mice untreated or infected with *H. felis* for 1 year. (**a**) Cystic gland dilatation. (**b**) Mucous cell metaplasia, arrow marks highlight mucous cells with foamy cytoplasm. (**c**) Gastric epithelial dysplasia. Individual animal scores and median plotted. ^Φ^signifies significant difference from untreated mice of same genotype. ^#^signifies significant difference from WT mice subjected to the same treatment, *P*<0.05. * indicates significant difference to WT animals, *P*<0.05. Scale bar 50 μm.

In humans, *H. pylori* infection results in distinct gastric epithelial damage either progressing towards gastric atrophy or peptic ulcer disease. In mice infected with *H. felis,* the most analogous early mucosal defect is cystic gland dilatation (Figure 8a), which is considered to be part of the inflammation-metaplasia-malignancy continuum in this species.^21^ *Nfkb1*^*−/−*^mice, but not other genotypes, exhibited spontaneous gland dilatation by 1 year. These lesions were significantly (*P*<0.05, two-way ANOVA on ranks) more frequent in infected *Nfkb1*^*−/−*^ animals. Dilated cystic glands also developed in WT and *c-Rel*^*−/−*^ mice following infection, but were absent from both infected and uninfected *Nfkb2*^*−/−*^ mice at this time point.

Glands exhibiting mucous cell metaplasia, characterized by the replacement of parietal cells with large foamy mucus secreting cells (Figure 8b, arrowed) staining positively with Alcian blue were quantified. Among uninfected WT mice, one exhibited a small number of glands with mucous metaplasia, as did two untreated *Nfkb1*^*−/−*^ mice, with no statistically significant differences between genotypes. Following long-term *H. felis* infection, mucous cell metaplasia was significantly more prevalent in WT, *Nfkb1*^*−/−*^and *c-Rel*^*−/−*^ mice compared with their uninfected counterparts. In contrast, *Nfkb2*^*−/−*^ mice had no increase in the number of glands demonstrating mucous metaplasia.

Spontaneous low-grade dysplastic lesions were observed in 66.7% of *Nfkb1*^*−/−*^ mice aged 1 year, but were not seen in other groups (*P*<0.05, Figure 8c). Following *H. felis* infection for 1 year, all groups apart from *Nfkb2*^*−/−*^ mice developed evidence of dysplasia, with the highest score being demonstrated in *Nfkb1*^*−/−*^ mice, but these differences in dysplasia scores did not reach statistical significance.

**Figure 9:**
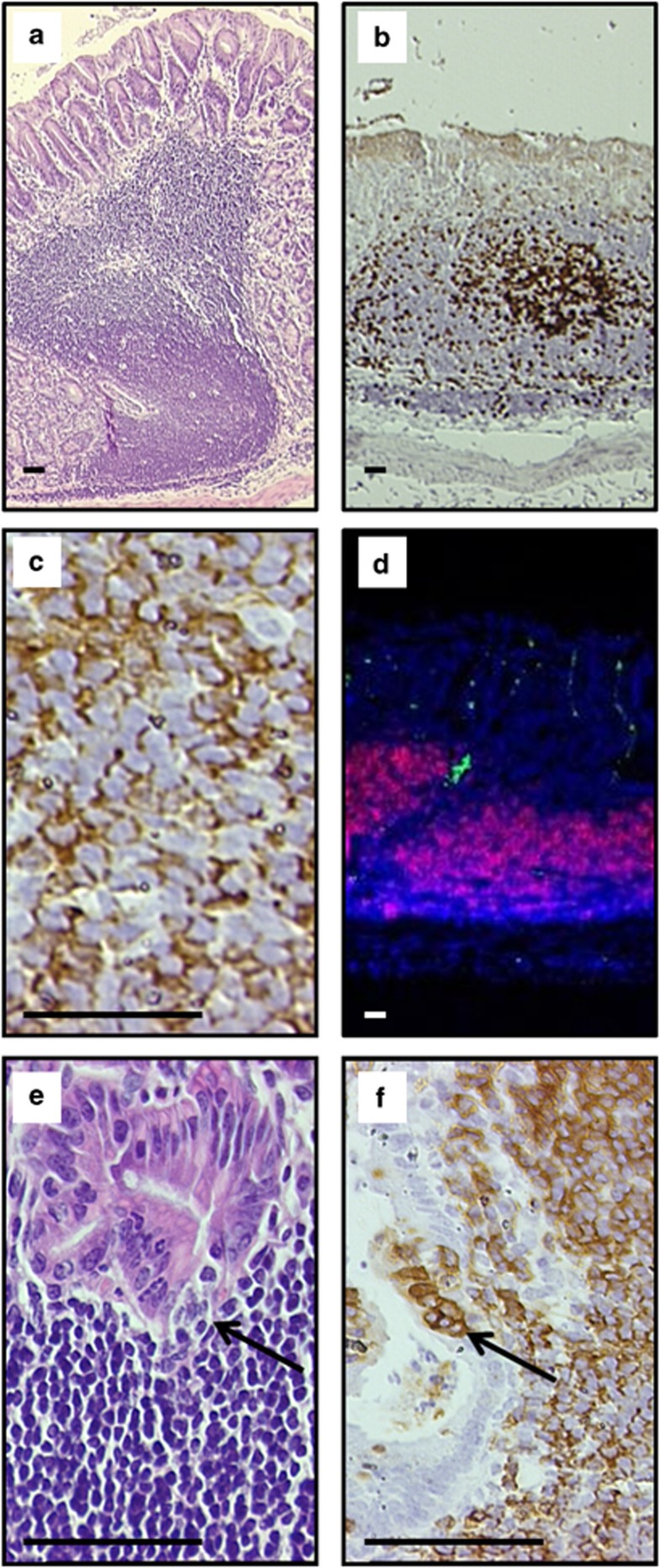
Disorganized lymphoid follicle formation in *H. felis-*infected *c-Rel*^*−/−*^mouse at 1 year, (**a**) H+E stained. (**b**) Ki67-positive staining of similar lesion. (**c**) CD45R-positive staining of lymphocytic infiltrates. (**d**) CD3 (green) CD20 (red) and DAPI (blue) co-staining showing B-cell predominance of lymphoid infiltrate. (**e**) H+E staining demonstrating cluster of lymphocytes encroaching on gastric gland, arrowed. (**f**) CD45R-positive cells encroaching on gastric gland, arrowed. Scale bar 50 μm.

In addition to the epithelial pathology described above, half (3 of 6) of the *c-Rel*^*−/−*^ mice exposed to *H. felis* for 1 year also developed lymphoproliferative lesions similar to, but less severe than the low-grade MALT lymphoma-like lesions identified in BALB/c mice by Enno *et al.*^22^ These lesions were characterized by marked gastric lymphoid hyperplasia, with large, highly proliferative (Ki67^+^ cells) and disorganized multifocal to coalescing lymphoid follicles (Figures 9a and b). The germinal centers of these lesions were disorganized or absent and bridging lymphoplasmacytic infiltrates were seen extending into the submucosa. Lymphocytes within the follicular structures and bridging infiltrates were almost entirely positive for the B-cell marker CD45R (80–95% of infiltrating lymphocytes) (Figure 9c), which was confirmed with CD20 and CD3 immunophenotyping, showing B-lymphocyte predominance with few T-lymphocytes present (Figure 9d). The inflammatory cell infiltrate also appeared to partially efface the mucosa; however, although occasional examples of CD45R-positive lymphocytes infiltrating into adjacent gastric glands were seen (Figures 9e and f), no established lymphoepithelial lesions were demonstrated.

## Discussion

Previous work has demonstrated that the deletion of NF-κB1 or NF-κB2 proteins can have different effects on gastrointestinal disease processes, including differences in the ability to clear *Trichuris* m*uris* parasites.^23^ Classical pathway NF-κB signaling has become one of the most studied pathways in inflammation-associated cancer; however, the function of specific NF-κB proteins has been relatively little studied in this context. We now demonstrate that signaling dependent on specific NF-κB proteins differentially affects the development of an inflammation-associated premalignant process.

In this study, *Nfkb1*^*−/−*^ mice developed the most pathology, with uninfected mice demonstrating spontaneous gastritis and gastric atrophy by 12 months of age. Previous *in-vivo* studies have described complex relationships between signaling mediated by NF-κB1 and inflammatory phenotypes. Deletion of NF-κB1 has been shown to protect from allergic airway inflammation,^24^ whereas NF-κB1^ΔC/ΔC^ mice, which constitutively overexpress transcriptionally active NF-κB1 p50, develop spontaneous hepatic and pulmonary inflammation in standard conditions.^25^ Conversely, colitis induced by intragastric administration of *Helicobacter hepaticus* was more severe in *Nfkb1*^*−/−*^ mice than WT,^26^ which is concordant with our observation of spontaneous gastric inflammation in mice of this genotype.

In humans, autoimmune atrophic gastritis is a risk factor for the development of gastric adenocarcinoma and neuroendocrine tumors.^18^ Similarly, other murine models that develop spontaneous gastric atrophy (for example, transgenically hypergastrinemic INS-Gas mice) also progress towards gastric cancer development more rapidly when exposed to *Helicobacter* infection.^27^ Our finding that, following short-term *H. felis* infection, *Nfkb1*^*−/−*^ mice also develop more severe gastric atrophy than mice of other genotypes was expected, but despite this there was no clear evidence of more rapid progression of other preneoplastic pathology in these mice.

In contrast, Shibata *et al.*^12^demonstrated that gastric epithelial cell deletion of inhibitor of κB kinase-β, and hence abrogation of the classical NF-κB pathway, induced a progressive phenotype of more severe atrophy and mucous cell metaplasia at 12 months. These findings suggest that signaling involving NF-κB1 acts as a negative regulator of inflammation and atrophy, but not mucous metaplasia.

Disruption of signaling involving NF-κB2 appears to be critically important for the development of gastric atrophy and other premalignant pathology in response to *H. felis* infection. Colonization scores and bacterial abundance were significantly increased in *Nfkb2*^*−/−*^ mice compared with WT, while inflammation scores and mucosal cytokine production were blunted 6 weeks after *H. felis* colonization. These findings coincide with scant epithelial pathology and raise the questions whether protection of the gastric epithelium after *Helicobacter* colonization is mediated by an impaired inflammatory response to *H. felis*, or whether there are also alterations in epithelial function that contribute to this. As animals with constitutively active NF-κB2 have been shown to develop gastric hyperplasia,^13^ it is likely that this NF-κB protein has a regulatory role on gastric mucosal proliferation. In support of this mechanism are our observations that demonstrate increased epithelial cell turnover together with enhanced processing of NF-κB2 P100 processing to P52 and DNA binding of NF-κB2 in *Nfkb1*^*−/−*^ mice. These findings are also supported by the observation that RelB is expressed within the gastric epithelium of WT mice exposed to long-term *H. felis* infection. Together these data support the hypothesis that signaling involving NF-κB2 and RelB are involved in regulating the consequences of *H. felis* infection in both epithelial and immune compartments of the gastric mucosa.

In *c-Rel*^*−/−*^ mice, gastric epithelial pathology indistinguishable from WT mice developed. Fifty percent of these animals also developed lymphoproliferative lesions morphologically similar to those described in Swiss mice by Ferrero *et al.*^28^ and in BALB/c mice by Enno *et al.*^22^ In the latter study, particularly severe pathology was observed with advanced lymphoepithelial lesions described as MALT lymphoma like. *c-Rel*^*−/−*^ mice did not develop lesions of this severity, but did progress to the stage described by Ferrero *et al.*^28^ Our investigations were performed on animals that were substantially younger than those studied by Enno *et al.,*^22^ and had been exposed to *Helicobacter* for less than half the duration of their studies, suggesting that c-Rel deletion may promote gastric lymphomagenesis in the context of *Helicobacter* exposure.

Few data have been published concerning the potential importance of c-Rel in *Helicobacter-*associated MALT lymphoma;^29^ however, c-Rel has been investigated in other B-cell malignancies. *In-vitro* overexpression of human *REL* acts oncogenically in a model of avian B-cell lymphoma,^30^ but not in murine or human B-lymphocyte culture. In diffuse large B-cell lymphoma, *REL* amplifications are common, being reported in approximately 15% of cases^31^ and overexpression of c-Rel is also associated with a worse prognosis.^32^ Other cases of B-cell lymphoma have been reported to be associated with the S525P *REL* mutation; *in-vitro* this mutation is more oncogenic than WT *REL*, but has reduced transcriptional activity.^33^ This may provide a biologically coherent mechanism to explain the enhanced B-cell lymphoid proliferation induced by *H. felis* following c-Rel deletion in our model.

In conclusion, these studies have demonstrated differential regulation of gastric pathology by signaling pathways involving NF-κB1, NF-κB2 and c-Rel. NF-κB2-mediated signaling appears to be required for the development of *H. felis-*induced gastric pathology; while NF-κB1-mediated signaling protects against spontaneous gastric inflammation and retards the progression of gastric atrophy, and possibly dysplasia, in response to *H. felis* colonization. Deletion of c-Rel potentially provides a novel model of gastric MALT lymphoma with three of six mice developing lymphoproliferative disease after 12 months of infection. This may provide direct insights into, and a means of studying, the pathogenesis of this important condition. To date, the significance of signaling mediated by NF-κB1, NF-κB2 and c-Rel has not been investigated in human gastric carcinogenesis. Our findings would suggest that this would merit further investigation.

## Materials and methods

### Animals

Transgenic animals were maintained on C57BL/6 genetic background, WT controls were sourced from Charles River (Margate, UK). Procedures were performed under UK Home Office licences at the University of Liverpool. Colonies of *Nfkb1*^*−/−*^,^20^ *Nfkb2*^*−/−*^^34^ and *c-Rel*^*−/−*^^19^ mice were maintained in conventional animal house conditions.

### Administration of Helicobacter to mice

*H. felis* ATCC:49179 were cultured as previously described^15^ and harvested into tryptone soy broth at a density of >10^9^ organisms/ml. Mice aged 6 weeks were administered 5 × 10^8^ bacterial on three occasions over 1 week.

### Quantitative histology

Scoring was performed blinded to genotype and treatment. Morphological features were scored by a European College of Veterinary Pathologists certified pathologist (JW). Haematoxylin and eosin (H+E) stains were used for morphology.

*H. felis* colonization was scored by visual analog scale defined as 0: no *Helicobacter* like organisms seen; 1: scanty *Helicobacter* seen in <5 glands per circumference; 2: either scanty *Helicobacter* seen in ⩾5 glands in one antral circumference OR heavy colonization of >5 antral glands in one circumference; 3: heavy colonization of ⩾5 antral glands in one circumference. Immune cell infiltration was scored using a previously described visual analog score.^35^

Gastric atrophy was assessed by measurement of mucosal thickness at 10 sites around the circumference of each section and calculating mean thickness. At each site the number of parietal cells was scored across a 10-μM wide tangential band through the mucosa.

Gastric corpus sections were immunolabeled for Ki67 (Dako, Ely, UK), cleaved caspase-3 (R&D systems, Minneapolis, MN, USA), NF-κB1 (Santa Cruz, Heidelberg, Germany) and RelB (Cell Signaling Technologies, Danvers, MA, USA). Antigen visualization was achieved using Dako secondary reagents. Ki67 staining was quantified as for parietal cells. Apoptosis was quantified by examining 10 high powered ( × 40 objective) fields per circumference.

Cystic gland dilatation was defined as a gland with dilated lumen and either necrotic debris within the lumen or attenuation of epithelial cells lining the gland. Glands were scored for mucous cell metaplasia only if they demonstrated both cells with foamy cytoplasm and Alcian blue positivity for greater than half the mucosal depth.

Lymphocyte typing was performed by immunohistochemistry for CD45R (AbCam, Cambridge, UK) and by co-immunofluorescence using goat anti-CD20 (Santa Cruz) and rabbit anti-CD3 (AbCam) primary antibodies.

### Real-time PCR

Nucleic acids were extracted from gastric tissues: DNA using DNEasy kit (Qiagen, Manchester, UK); RNA by HighPure Tissue RNA kit (Roche, Burgess Hill, UK). Samples were normalized to *Gapdh* and are represented as linearized fold changes as calculated by the Genex software package (MultiD Analyses AB, Gothenburg, Sweden). Cycling conditions, primers and probes are shown in Table 1.

### Western blotting

Western blotting was performed using anti-RelA (Santa Cruz), anti-RelB (Cell Signaling Technologies), anti-NF-κB1 (Santa Cruz), anti-NF-κB2 (Cell Signaling Technologies), anti-NIK (Santa Cruz), anti-panactin and anti-cyclin D1 primary antibodies (both Millipore, Watford, UK).

### DNA-binding enzyme-linked immunosorbent assay

Nuclear extracts were generated by sucrose gradient extraction and were subjected to a proprietary DNA-binding enzyme-linked immunosorbent assay(TransAM NF-κB2 p52, ActiveMotif, Rixensart, Belgium).

### Statistics

Normally distributed data were analyzed by one- and two-way ANOVAs with Holm–Sidak *post-hoc* analyses. Where data were heteroscedastic, two-way ANOVAs were performed on ranked data. Visual analog scores were treated as ordinal and assessed by Kruskal–Wallis one-way ANOVA and Dunn's *post-hoc* analysis.

## Figures and Tables

**Table 1:** Primer and probe designs and real-time PCR conditions

*Target*	*Forward primer*	*Reverse primer*	*UPL probe*
GAPDH	5′-ggg-ttc-cta-taa-ata-cgg-act-gc-3′	5′-cca-ttt-tgt-cta-cgg-gac-ga-3′	No. 52
FlaA	5′-gtt-tct-ggg-gcc-aat-gta-ag-3′	5′-gcc-ttt-ggc-aaa-acc-aat-ag-3′	No. 120
TNF-α	5′-tct-tct-cat-tcc-tgc-ttg-tgg-3′	5′-ggt-ctg-ggc-cat-aga-act-ga-3′	No. 49
IL-1β	5′-ttg-acg-gac-ccc-aaa-aga-t-3′	5′-gaa-gct-gga-tgc-tct-cat-ctg-3′	No. 26
IFN-γ	5′-atc-tgg-agg-aac-tgg-caa-aa-3′	5′-ttc-aag-act-tca-aag-agt-ctg-agg-ta-3′	No. 21
Fas	5′-tgc-aga-cat-gct-gtg-gat-ct-3′	5′-ctt-aac-tgt-gag-cca-gca-agc-3′	No. 34
FasL	5′-acc-ggt-ggt-att-ttt-cat-gg-3′	5′-agg-ctt-tgg-ttg-gtg-aac-tc-3′	No. 21

*Cycle*	*Temperature (°C)*	*Duration (seconds)*	*Cycles*
*Thermal cycler conditions*			
Pre-incubation	95	600	1
Amplification	95	10	45
	60	30	
	72	1	
Cooling	40	30	1

Abbreviations: IFN-γ, interferon-γ IL-1β, interleukin-1β TNF-α, tumor necrosis factor α

## Supplementary Material

Supplementary Figure1
