# Supplementary figures and images for: Signaling mediated by the NF-κB sub-units NF-κB1, NF-κB2 and c-Rel differentially regulate Helicobacter felis-induced gastric carcinogenesis in C57BL/6 mice
Source: Oncogene. 2013 Aug 26;32(50):5563–73. doi: 10.1038/onc.2013.334 (PMC3898319; doi:10.1038/onc.2013.334)

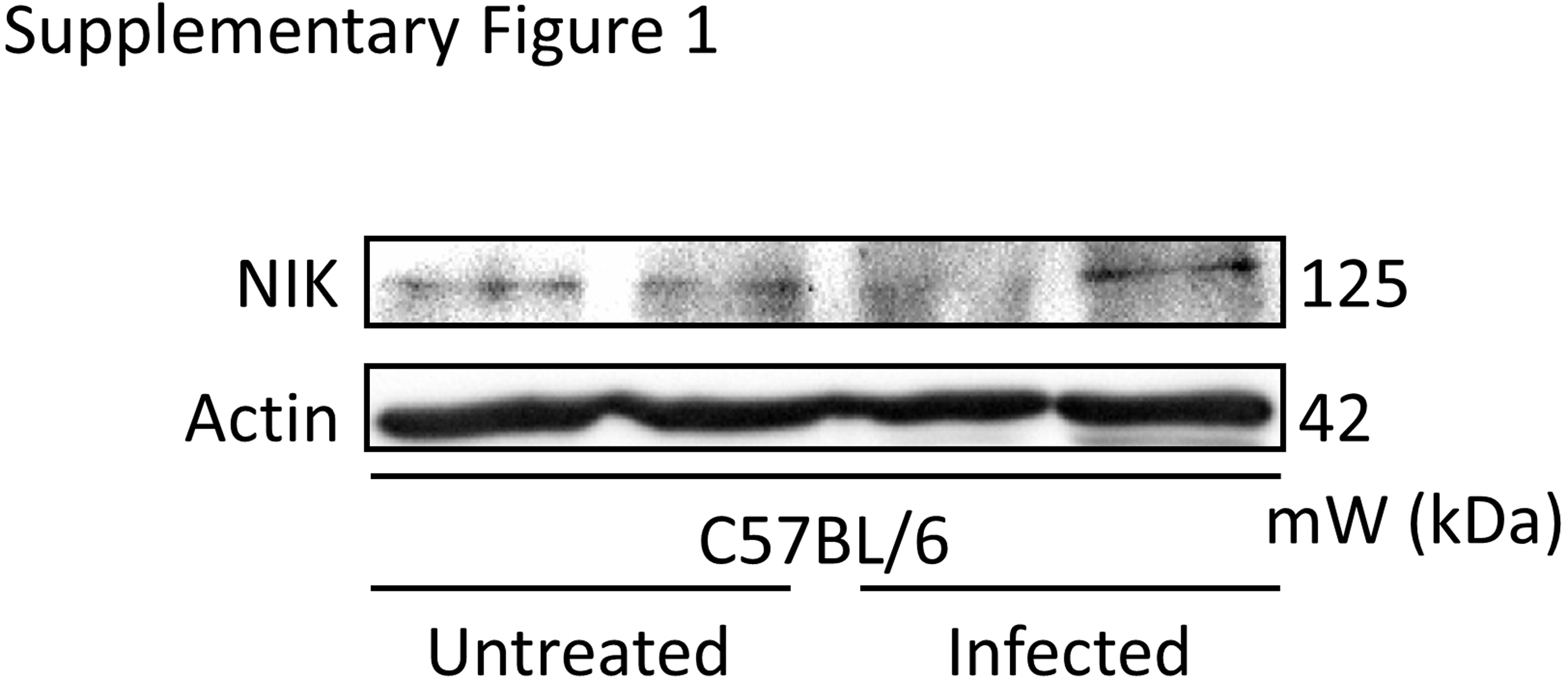

Supplement: Supplementary Figure1 [file onc2013334x1.tif]
