# Supplementary material for: Signaling mediated by the NF-κB sub-units NF-κB1, NF-κB2 and c-Rel differentially regulate Helicobacter felis-induced gastric carcinogenesis in C57BL/6 mice
Source: Oncogene. 2013 Aug 26;32(50):5563–73. doi: 10.1038/onc.2013.334 (PMC3898319; doi:10.1038/onc.2013.334)
Supplement: Supplementary Information [file onc2013334x2.doc]

**Supplementary Figure Legend**

Supplementary Figure 1: Western blot demonstrating expression of NIK in gastric corpus of WT mice either untreated, or infected with *H. felis* for 6 weeks, actin blot used as loading control.

**Supplementary table 1:** Summary of Histopathological scoring in long term *H. felis* infection

| Genotype | Treatment | Inflammation Score | Cystic Glands | Mucous Metaplasia | Dysplasia |
| --- | --- | --- | --- | --- | --- |
| C57BL/6 | Untreated | 0 | 0 | 27 | 0 |
| C57BL/6 | Untreated | 0 | 0 | 1 | 0 |
| C57BL/6 | Untreated | 0 | 0 | 1 | 0 |
| C57BL/6 | Untreated | 0 | 1 | 1 | 0 |
| C57BL/6 | Untreated | 0 | 0 | 1 | 0 |
| C57BL/6 | Untreated | 2 | 1 | 1 | 0 |
| *Nfkb1^-/-^* | Untreated | 3 | 5 | 1 | 0 |
| *Nfkb1^-/-^* | Untreated | 3 | 2 | 1 | 0 |
| *Nfkb1^-/-^* | Untreated | 3 | 1 | 34 | 1 |
| *Nfkb1^-/-^* | Untreated | 3 | 4 | 1 | 1 |
| *Nfkb1^-/-^* | Untreated | 3 | 1 | 31 | 1 |
| *Nfkb1^-/-^* | Untreated | 3 | 0 | 1 | 1 |
| *Nfkb2^-/-^* | Untreated | 0 | 0 | 1 | 0 |
| *Nfkb2^-/-^* | Untreated | 0 | 0 | 1 | 0 |
| *Nfkb2^-/-^* | Untreated | 0 | 0 | 1 | 0 |
| *Nfkb2^-/-^* | Untreated | 0 | 0 | 1 | 0 |
| *Nfkb2^-/-^* | Untreated | 0 | 0 | 1 | 0 |
| *Nfkb2^-/-^* | Untreated | 0 | 0 | 1 | 0 |
| *c-Rel^-/-^* | Untreated | 0 | 0 | 1 | 0 |
| *c-Rel^-/-^* | Untreated | 0 | 0 | 1 | 0 |
| *c-Rel^-/-^* | Untreated | 0 | 0 | 1 | 0 |
| *c-Rel^-/-^* | Untreated | 0 | 0 | 1 | 0 |
| *c-Rel^-/-^* | Untreated | 0 | 0 | 1 | 0 |
| *c-Rel^-/-^* | Untreated | 0 | 0 | 1 | 0 |
| *c-Rel^-/-^* | Untreated | 0 | 1 | 1 | 0 |
| *c-Rel^-/-^* | Untreated | 0 | 0 | 1 | 0 |
| C57BL/6 | Infected | 3 | 1 | 35 | 1 |
| C57BL/6 | Infected | 3 | 4 | 32 | 1 |
| C57BL/6 | Infected | 3 | 15 | 43 | 2 |
| C57BL/6 | Infected | 3 | 12 | 42 | 0 |
| C57BL/6 | Infected | 2 | 0 | 33 | 0 |
| C57BL/6 | Infected | 3 | 46 | 37 | 1 |
| C57BL/6 | Infected | 3 | 24 | 41 | 2 |
| *Nfkb1^-/-^* | Infected | 3 | 15 | 25 | 2 |
| *Nfkb1^-/-^* | Infected | 3 | 10 | 45 | 2 |
| *Nfkb1^-/-^* | Infected | 3 | 15 | 44 | 1 |
| *Nfkb1^-/-^* | Infected | 3 | 23 | 39 | 2 |
| *Nfkb1^-/-^* | Infected | 3 | 6 | 26 | 1 |
| *Nfkb2^-/-^* | Infected | 2 | 0 | 29 | 0 |
| *Nfkb2^-/-^* | Infected | 0 | 0 | 1 | 0 |
| *Nfkb2^-/-^* | Infected | 2 | 0 | 1 | 0 |
| *Nfkb2^-/-^* | Infected | 0 | 0 | 1 | 0 |
| *Nfkb2^-/-^* | Infected | Died 6/12 post H felis infection – normal stomach | | | |
| *Nfkb2^-/-^* | Infected | Died 8/12 post H felis infection – normal stomach | | | |
| *c-Rel^-/-^* | Infected | 3 | 29 | 38 | 2 |
| *c-Rel^-/-^* | Infected | 3 | 26 | 30 | 2 |
| *c-Rel^-/-^* | Infected | 3 | 5 | 28 | 0 |
| *c-Rel^-/-^* | Infected | 3 | 9 | 36 | 1 |
| *c-Rel^-/-^* | Infected | 4 | 38 | 46 | 1 |
| *c-Rel^-/-^* | Infected | 3 | 0 | 1 | 2 |
| *c-Rel^-/-^* | Infected | 3 | 12 | 40 | 2 |
